# Methyl iodide poisoning presenting as a mimic of acute stroke: a case report

**DOI:** 10.1186/1752-1947-4-177

**Published:** 2010-06-15

**Authors:** Jagdish R Nair, Kausik Chatterjee

**Affiliations:** 1 Department of Medicine and the Acute Stroke Unit, Countess of Chester Hospital, Chester, UK

## Abstract

**Introduction:**

Stroke mimics are usually non-vascular disease processes. These raise the possibility of a stroke and are common in clinical practice. It is necessary to distinguish these mimics in order to provide early and appropriate management, as well as reduce possible harm on our patient.

**Case presentation:**

We report the case of a 50-year-old Caucasian man who developed symptoms suggestive of posterior circulation stroke after he was exposed to methyl iodide at his workplace. Results of stroke investigations of our patient were negative, and a detailed occupational history clinched the diagnosis. Acute presentation with a stroke-like picture is rare in cases of methyl iodide poisoning. We have attempted to discuss the differential diagnosis of stroke mimics through a review of literature.

**Conclusion:**

Stroke mimics are difficult to diagnose in an emergency room situation and may be initially treated as stroke. This case report underlines the importance of history taking, especially occupational history, in the differential diagnosis of stroke. We also stress the need to recognize mimics at presentation in order to arrive at an early and appropriate management of patients.

## Introduction

Several conditions raise the suspicion of a stroke, but there usually are alternate explanations for their symptoms. These conditions are commonly known as "stroke mimics". Nearly a third to half of cases referred to hospitals as suspected strokes turn out as stroke mimics [1]. Here, we present the case of a 50-year-old man who presented signs of as a stroke mimic. In addition, we briefly discuss the various known causes of stroke mimics.

## Case presentation

A 50-year-old Caucasian man, who was a chemical factory worker, presented to our accident and emergency department, Countess of Chester Hospital, Chester, with sudden onset of slurred speech, double vision, heaviness on the right side of his body and unsteadiness of gait. These symptoms had started two days prior to his admission. He also reported having headaches, which got worse with time.

On admission, his vital signs were at a normal range except for an elevated blood pressure (BP) of 180/60 mmHg and raised temperature of 38°C, which stabilized subsequently. He also had some shortness of breath and an initial non-productive cough, but his condition had improved at the time of his presentation to the hospital. Neurological examination showed a Glasgow Coma Scale (GCS) of 14 to 15, a bilateral grade 2 nystagmus, slurred speech, past pointing on the right, and a broad-based ataxic gait. However, he demonstrated no objective motor weaknesses or sensory deficits. An examination of his chest, cardiovascular and gastrointestinal systems were unremarkable. In view of the above history and clinical findings, a diagnosis of cerebrovascular accident (CVA), possibly involving the vertebrobasilar territory, was made and was further investigated.

Investigations included computed tomography (CT) and magnetic resonance imaging (MRI) of his brain, which all yielded normal results. A routine blood investigation including full blood count, renal functions, liver function tests and thyroid functions also showed normal results. His lipid profile showed raised random cholesterol (6.2 mmol/L) and triglycerides (2.68 mmol/L) levels. Cerebrospinal fluid (CSF) studies performed on our patient in view of his fever and neurological symptoms were normal.

Due to these negative test results and the presence of a small alkali burn on the extensor aspect of his right wrist, our patient's occupational history was considered. He had recently started working with methyl iodide and had spillage of the chemical through his suit and on to his hand a week before the onset of his symptoms. His wife subsequently reported that he was sleepier and less interactive. He also complained of visual and auditory hallucinations.

We discussed our patient's case with the National Poisons Center and they suspected that his symptoms were due to methyl iodide poisoning. He was observed on the ward and discharged on the third day with an advice to stay off work until the neurological symptoms had cleared. He was reviewed in out-patient clinic two weeks later and was noted to have made an improvement in hallucinations and slurred speech. He was, however, still ataxic. Subsequent review at six weeks revealed an improvement in his cognition and ataxia. At three months, he showed only minimal deficits in coordination.

## Discussion

A diagnosis of stroke by a non-stroke physician can sometimes be a challenge. Studies have shown that nearly a third to half of our patients referred by general practitioners or emergency physicians as having a suspected stroke are actually exhibiting stroke mimics [1]. These are primarily non-vascular disease processes producing some of the clinical pictures akin to stroke (Table 1).

**Table 1:** Non-vascular disease processes producing some of the clinical picture akin to a stroke

Conditions	Comments
**A. Nervous system**	

Epilepsy	Todd's palsy presents with postictal confusion, transient focal motor and sensory symptoms, extra-ocular movement deficits; witness and/or history is important and has a resolving course.

Hemiplegic migraine [4]	Headache, impaired consciousness, ataxia and hemi paresis; stereotypical attacks can occur.

Infection(meningitis/encephalitis/abscess)	Headache, hemiparesis, altered consciousness; systemic disturbances common

Intracranial mass lesions [14]	Subdural hematoma, tumors and abscesses can present with headache and abrupt onset focal neurological symptoms. Fever, weight loss, systemic disturbances are common.

Encephalopathy	Can present with altered behavior, cognition, coma, aphasia, focal motor and sensory deficits, homonymous hemianopia and hemi hyperreflexia.

Multiple sclerosis	Visual and long tract neurological symptoms; recurrences of neurological deficits in space and time are common.

Myasthenia gravis (rare)	Ocular myasthenia and weakness mimic stroke; variable and diurnal fluctuation.

**B. Non-nervous system**	

Syncope	Usually due to hypotension or arrhythmia; Vertebrobasilar insufficiency can cause syncope.

Sepsis	Various neurological presentations, systemic disturbances common

Hyponatremia, hyperglycemia, hypoglycemia [5]	Can present with fluctuating neurological symptoms, aphasia, cortical blindness and so on; an initial blood sugar check is vital.

Functional disorders	Conversion disorders can have hemiparesis, blindness, speech disturbances; psychiatric history

Industrial toxins, drug overdose	Conversion disorders can have hemiparesis, blindness, speech disturbances; psychiatric history

Among the various neurological conditions mimicking strokes, seizures are the most common at 23% [2]. Postictal neurological deficits (also known as Todd's palsy) may be difficult to interpret specifically if seizures were unwitnessed. Usually, however, these deficits have accompanying lethargy and, in majority of cases, neurological deficits resolve completely. Also, it is rare (< 1%) that seizures occur as a presentation of stroke, which lends it difficult to differentiate from Todd's palsy.

Both intracranial (encephalitis and meningoencephalitis) and extracranial sepsis, particularly in elderly patients, can present as a stroke mimic. In these cases, prompt identification of intracranial sepsis is important, as any delay in instituting antibiotic or antiviral treatment may produce grave consequences. In most series, the median delay in diagnosing herpes simplex encephalitis from symptoms to presentation was reported as 5.5+/to 2.9 days [3]. This avoidable delay contributes significantly in obtaining a poor outcome in treating patients. Meanwhile, hemiplegic and ocular varieties of migraine can also manifest as a stroke [4], but migraine usually has a recurrent stereotype presentation.

Among the non-neurological stroke mimics, syncope is the most common at 23% of reported cases [2]. Hypoglycemia may sometimes present with fluctuating neurological symptoms, and cortical blindness and aphasia have been reported in patients with hypoglycemia [5].

On the other hand, poisoning seldom presents as a stroke mimic. Due to its limited availability, methyl iodide poisoning is rarely seen in clinical practice, and only fewer than 15 case reports have been described in the literature.

Methyl iodide is a monohalomethane, analytic and organic chemistry reagent used by the pharmaceutical industry in microscopy for refraction and also as a fumigant. The population at risk of methyl iodide poisoning via inhalation are workers at the industry, such as occupational groups like tractor drivers, shovelers, soil sealers and tarp removers involved in pre-plant field fumigation. Bystanders (general population) near the area of fumigation are also at risk. The risk through water absorption and to those in the pharmaceutical industry, however, is not significant.

The exact mechanism of neurotoxicity is still debated on, but glutathione (GSH) depletion through methyl halide metabolism is hypothesized. It is also believed that neurotoxicity develops through the high lipid solubility of methyl iodide and due to the effects of proteins and macromolecules on methylation [6].

In an acute exposure, methyl iodide is a pulmonary and dermal irritant, causing pulmonary edema and alkali burn. Characteristics of the poisoning are: delay in the onset of symptoms after an exposure to the chemical; renal failure, cerebellar and Parkinsonian symptoms, seizures and coma occurring in severe cases; and psychiatric disturbances like personality changes (sleeping problems, excitation, depression, delusions and hallucinations) and cognitive problems (memory, learning, language and cognitive reaction problems) that can last for months or even years [7].

A review of the documented cases shows that many patients had experienced chronic neurological syndromes characterized primarily by delayed psychiatric, behavioral and cognitive sequelae [8]. Some patients had purely psychiatric symptoms, while others had neurological deficits as well. Some patients had recovered fully in a course of months, while others had persistent symptoms. Our patient had a fairly rapid onset of neuropsychiatric symptoms presenting like a posterior circulation stroke. The acute onset could have been related to a large accidental exposure via inhalation of the chemical, although the quantity of such exposure is usually difficult to determine. An interesting laboratory marker, although non-specific, is a rise in lipid level as was observed in our patient [9].

Other poisons that may behave like methyl iodide in its neurotoxicity include monohalomethanes like methyl chloride and bromide, solvents like toluene (glue sniffing) and insecticides like organophosphates. The mechanism of toxicity is different for toluene and organophosphates. Cases of poisoning usually involve those who are part of the production and handling of these chemicals.

The accurate diagnosis of stroke depends on how accurate is our patient history obtained using clinical signs suggestive of an anatomical lesion in the part of the brain supplied by a blood vessel. Recent advances in imaging techniques, such as diffusion-weighted MRI, is accurate in supporting the diagnosis [10] in most of the cases reported. However, there is still no substitute for proper history taking and clinical examination.

A recent study analyzing the factors that help in distinguishing mimics from a stroke showed that impairment in cognition and disturbances in other systems are more likely to suggest a mimic. On the other hand, the exact time of onset, which lateralized focal symptoms and signs, suggested a stroke [11]. Another study suggested that an acute onset of disorders of language function is suggestive of cerebrovascular disease, but if associated with changes in behavior may instead suggest a non-stroke physiology [12]. Other studies have shown that reduced levels of consciousness and normal extra-ocular movements favor a mimic, while abnormal extra-ocular movements, visual fields, hypertension, arrhythmias and vascular risk factors reduce the chances of a mimic [13]. Thus, clinical examination provides us with a strong tool to differentiate mimic from a true stroke.

## Conclusions

A rapid and accurate diagnosis of stroke using clinical assessments aided by imaging techniques is pivotal to the early treatment through thrombolysis, reversal of anticoagulation, and surgical intervention for malignant middle cerebral artery (MCA) syndrome. Such prompt and correct diagnosis would reduce brain injury during the acute phase of the stroke. Stroke mimics can cause diagnostic dilemma and clinicians should be aware of the possibility of such presentations.

Methyl iodide poisoning is rare in clinical practice. It can mimic an acute stroke or a neuropsychiatric condition. Diagnosis is made clinically and management is supportive. Its recognition in this case was important as our patient could have been inappropriately treated with anti-platelet agents or psychiatric drugs, and the issue of addressing a change in occupational environment might have been missed.

## Abbreviations

BP: blood pressure; CXR: chest X-ray; CRP: C-reactive protein; CVA: cerebrovascular accident; CT: computed tomography; CSF: cerebrospinal fluid; FBC: full blood count; GSH: glutathione; LFT: liver function test; MRI: magnetic resonance imaging.

## Consent

Written informed consent was obtained from our patient for publication of this case report and any accompanying images. A copy of the written consent is available for review by the Editor-in-Chief of this journal.

## Competing interests

The authors declare that they have no competing interests.

## Authors' contributions

JRN made contributions to the design, acquisition of data, literature review and drafting of the manuscript. KC was responsible for the design, conception, drafting and supervision of this work. Both authors read and approved the final manuscript.
